# Home Mechanical Ventilation: A Patient’s Perspective Survey Study

**DOI:** 10.3390/ijerph18084048

**Published:** 2021-04-12

**Authors:** Magdalena Kwiatosz-Muc, Bożena Kopacz

**Affiliations:** Department of Aneasthesiological and Intensive Care Nursing, Medical University of Lublin, 20-093 Lublin, Poland; bozenakopacz@umlub.pl

**Keywords:** advanced nursing, health care quality, home mechanical ventilation, patient’s satisfaction

## Abstract

Background: An increasing number of patients included in home mechanical ventilation (HMV) care has been under observation for many years. The study aimed to assess the patients opinion concerning the expected and perceived quality of care in an HMV system and a patient’s satisfaction with care. Methods: In 2017, patients treated with HMV were surveyed in Poland with the modified SERVQUAL questionnaire. Results: One hundred correctly completed surveys were analyzed. Patient Satisfaction Index was high. In every examined area, the expectations were statistically significant larger than the perception of the services. The biggest gap was in the tangibility dimension and the smallest gap was in the empathy dimension. Perceived respect and understanding for a patient’s needs are close to the expectations. Conclusions: The level of satisfaction with health care among patients treated with HMV in majority of investigated components is high. Moreover, the difference between perceived and expected quality of health care in the HMV system was relatively small in the opinion of the patients themselves. Further investigations with alternative methods are needed.

## 1. Introduction

An increasing number of patients included in home mechanical ventilation (HMV) care has been under observation for many years [1,2,3,4,5]. HMV is a complex and multidisciplinary treatment for patients with symptoms of chronic respiratory insufficiency due to pulmonary disorders, scoliosis or neuromuscular disorders. Treating these patients with home mechanical ventilation programs decreases the frequency of visits in emergency departments and hospitalizations in general [6]. Several studies have shown increased survival among HMV patients [4,7,8]. Recent guidelines of several scientific associations recommend the use of HMV for patients, with symptoms of chronic respiratory insufficiency [9,10,11,12,13].

The number of patients treated with ventilation at home has been increasing constantly. The prevalence of HMV differs greatly in different parts of the world [14] The prevalence of HMV in Canada is 12.9 patients/100,000 [15,16]. In Australia and New Zealand, these values are 9.9 and 12.0 patients/100,000 accordingly [16]. The most current data from East-Central Europe come from Hungary and show that the prevalence of HMV is 3.9 patients/100,000 [14].

The experiences of caregivers of patients undergoing HMV have been described in the literature [15], as well as the experiences of the patients themselves [16,17,18]. These experiences are related to the psychological functioning of these patients [19], as well as to the health-related quality of life [20,21,22]. Very few studies describe the patient’s perspectives concerning the system of home mechanical ventilation.

The measurement of the quality of medical services and patient’s satisfaction is performed in many medical disciplines. However, HMV is very rarely assessed in this perspective. The association between medical service quality and patient’s satisfaction was already suggested by other researchers [17,18]. Nevertheless, the process of assessment of the patient’s perspective of quality of health care services has been a challenge for years [17], especially if the care is delivered at home [19]. Until now, the best way to measure its quality in medicine has not been defined [17,19,20]. The SERVQUAL gap model and importance performance analysis (IPA) are used in the measurement of quality of services [17,21,22]. SERVQUAL, originally used in the measurement of quality in production units, was also used in medical services in order to measure a patient’s opinion concerning nurse care [23,24], specialist ambulatory health care [21], and other medical branches [25,26,27]. The SERVQUAL model was designed in 1985 by Parasurman et al. and allows measuring the quality of services according to a tested person’s opinion by measuring the differences between the quality expected and the quality perceived [28]. This difference may be recognized as the measure of satisfaction with the received services [18].

To analyze the HMV system in the aspect of its functioning, its patients and their feeling of safety would be very valuable. The satisfaction assessment of patients with HMV services related to perception of the quality of medical services in the HMV system would be a source of an important piece of information, which is helpful in the optimization of system functioning.

## 2. Methods

The study aimed to assess the expected and perceived quality of care in an HMV system and assess the patient’s satisfaction with care. An attempt was undertaken to find these areas of care for HMV patients, which could be improved to increase the patient’s satisfaction.

In 2017, patients treated with HMV were surveyed in the South-East of Poland. Only adult patients were included in the study, with the length of HMV treatment being between 6 months and 10 years. The modified SERVQUAL questionnaire was used. The authors surveyed the relevant sociological and demographic data combined with a medical history. A total of 125 surveys were distributed by the members of the research team to the caregivers of the patients. Surveys were left and completed without the presence of the member of the therapeutic team. The survey in the envelope was taken by the next visit. Finally, the envelope with the survey was passed to the research team. The traditional form of a paper and pencil survey was used.

The classic SERVQUAL questionnaire is composed of 22 items in five dimensions (tangibility, reliability, responsiveness, confidence, and empathy) [17,21]. In this study, a modified questionnaire was used. In every dimension, the questionnaire was composed of variables specific to the medical services delivered to the patient’s home. In the classic SERVQAL questionnaire, the visual appearances of facilities in the place of service giving and modern equipment are assessed. If the care is delivered at the patient’s home, the service giver has no influence on this area. As a result, in the tangibility dimension, only the material status of the participants was assessed. In the reliability dimension, the accuracy of medical services was assessed, as well as a will to help the patient and their family in solving medical problems. Keeping calm and the level of care were assessed, as well. In the responsiveness dimension, the rapidity in the response to the patient’s requests was assessed. In the confidence dimension, the safety of the patient as well as the courtesy and competence of personnel were assessed, along with the availability of a physician, nurse, and physiotherapist. In the empathy dimension, the individual attitude to a patient was assessed: respect and understanding for the patient’s needs, intimacy preservation, way of passing on information, and ability to listen to the patient. Each of the aspects was assessed by the respondents in the expectation perspectives and received services perspective. A five-degree (1 to 5) Likert scale was used. The modified SQRVQUAL questionnaire used in the study is provided in the Supplementary Material (Table S1). For every question and dimension, the gap between the perceptions and expectations was calculated.

With five dimensions: tangibility, reliability, responsiveness, confidence, and empathy patients (PSI) satisfaction index (with 100% maximum) was calculated by the formula (Equation (1)):

(1) $$PSI=\frac{{\bar{x}}_{1}}{\max x_{1}}\cdot 20\%+\frac{{\bar{x}}_{2}}{\max x_{2}}\cdot 20\%+\frac{{\bar{x}}_{3}}{\max x_{3}}\cdot 20\%+\frac{{\bar{x}}_{4}}{\max x_{4}}\cdot 20\%+\frac{{\bar{x}}_{5}}{\max x_{5}}\cdot 20\%$$

when:

where ${\bar{x}}_{i},\;i=1,\ldots ,5$ represents mean value of the current dimension and$\;\max x_{i},\;i=1,\ldots ,5$ represents the maximum values of this dimension.

In this study, the tangibility dimension of the material status of the participants was assessed. The service giver has no influence. This is why, with four dimensions: reliability, responsiveness, confidence, and empathy, the patient’s (PSI) satisfaction index (with 100% maximum) was also calculated using the following formula (Equation (2)):

(2) $$PSI=\frac{{\bar{x}}_{1}}{\max x_{1}}\cdot 25\%+\frac{{\bar{x}}_{2}}{\max x_{2}}\cdot 25\%+\frac{{\bar{x}}_{3}}{\max x_{3}}\cdot 25\%+\frac{{\bar{x}}_{4}}{\max x_{4}}\cdot 25\%$$

where ${\bar{x}}_{i},\;i=1,\ldots ,4$ represents the current dimension and$\;\max x_{i},\;i=1,\ldots ,4$ represents the maximum values of this dimension.

### Statistical Methods

The data were analyzed statistically using the STATISTICA 13.3 program Tibco Software (Inc. Palo Alto, Ca, USA). The qualitative variables were characterized by multiplicity and percentage, while the quantitative variables were characterized by basic classical statistical measures: mean and standard deviation. If the features were far from the normal distribution, the positional measure of the average value—median and quartile intervals Q1–Q3—were used. The scope of minimal and maximal was given. The Wilcoxon test for two related samples was used and the ANOVA Kruskal–Wallis was used for a larger amount of independent samples.

## 3. Results

One hundred and twenty-five surveys were distributed among the HMV patients. Only 100 correctly completed surveys were included in the analysis. Twenty-one surveys were incomplete. The mean age in the examined population was 66 (Q1–Q3; 57–72). The examined population included 71 (71%) patients with pulmonary diseases. Among these patients, 65 were diagnosed with COPD (chronic obstructive pulmonary disease), two with bronchial asthma, two with apnea syndrome, one person with amyloidosis, and one with pulmonary emphysema. Twenty-nine (29%) of patients from the entire examined population had respiratory insufficiency due to neuromuscular disorders (NMD), such as ALS (amyotrophic lateral sclerosis) (13 persons), MD (muscular dystrophy) (six persons), myasthenia gravis (four persons), SM (sclerosis multiply) (two persons), cerebral palsy (two persons), central core disease (one person), and Arnold–Chiari Syndrome (one person). Twenty-nine patients from the examined population were treated with IV (invasive ventilation). The characteristics of the examined population are shown in detail in Table 1.

The calculation of the patient satisfaction index (PSI) for all five dimensions is high: at 87.16%. The calculation of the patient satisfaction index (PSI) for four dimensions (without tangibility) is 91.2%.

In every examined area, the expectations were statistically important and larger than the perceptions of the services. The biggest gap was in the tangibility dimension. It reflects the material status of the family. Here, the difference between the expectations and perceptions is largest in all dimensions (Table 2 and Table 3).

The smallest gap (however statistically important) between the expectations and perceptions was in the empathy dimension. The perceived respect and understanding for the patient’s needs (gap −0.18, *p* < 0.001 and −0.19, *p* < 0.001 accordingly) are close to the expectations. The perceived intimacy preservation and way of passing on information, as well as the ability to listen to the patient (gap −0.21, *p* < 0.001 and −0.23, *p* < 0.001, accordingly) are also close to the expectations. In the confidence dimension, the difference between the expectations and perceptions was relatively small (−0.27, *p* < 0.001). In particular, the competence of personnel, the availability of the nurse, and courtesy of the personnel were close to the patient’s expectations. A comparison of the expected and perceived quality of care is shown in Table 2. In Table 3, a comparison of the expected and perceived quality of care in particular dimensions is shown.

A distribution of values in particular dimensions divided by groups created by demographical variables (gender, marital status, level of education, place of living, as well as living conditions) was performed. Respondents with bad and very bad living conditions showed a significantly lower level of satisfaction than the tangibility dimension compared to people with good and very good conditions (Table 4).

No significant differences were found in the satisfaction with the dimension of reliability, responsiveness, confidence, and empathy, depending on gender, marital status, level of education, and place of living.

## 4. Discussion

The seldom-described experiences of patients undergoing HMV show that this treatment causes distress and anxiety in the patients’ lives [29], as they have a constant feeling of dependence. In the German study of Schaepe and Ewers, one of the respondents described the total necessity of trusting one’s nurse [30], which reflects this high feeling of dependence. Similar feelings of patients concerning their caregivers were described in a Scottish study by MacLaren et al. [31].

The reflections of a patient who is treated with HMV in the context of satisfaction with the received services were investigated only vary rarely. In the study from Taiwan, the satisfaction of HMV patients was assessed with the SERVQUAL method. The authors of this study described very small gaps between the level of expectations and perceived quality in every examined dimension [17]. It reflects a high satisfaction of patients with the received medical services, which correlates with the results of our study. In the Taiwan study, the gap in the tangibility dimension is smaller than in the present study. Here, this dimension reflects the material and living conditions of respondents. However, patients in the Taiwan study were treated in health care institutions.

The functioning of the HMV system is rarely investigated and has not been examined in Poland. The HMV services in Poland are delivered in the patient’s place of living [2]. There are many health care providers in the HMV system in Poland, which are all covered by a public payer (National Health Fund) [2]. Ventilators, oxygen concentrators, and respiratory care equipment are delivered by the health care providers at the cost of the taxpayer. The nurse, physiotherapist, and physician care takes place in the patient’s home and is provided by the health care provider. The physician is a specialist of anesthesiology, pulmonology or neurology. The frequency of follow up depends on the type of ventilation (NIV or IV). The IV patients are supervised more often than the NIV patients. The NMD patients are followed up more often than the COPD patients (similarly to the IV patients and NIV patients).

The calculated PSI for five and four dimensions are high (91.2% and 87.16% accordingly). However, there are significantly statistical differences in the expected and perceived components of care. Moreover, in the present study, patients’ expectations concerning care are close to the perceived quality of care in the majority of assessed components. It reflects patient’s satisfaction with the care system. It reflects the high quality of health care in this area of medicine. 

The biggest gap between the expectations and the perceptions of quality was found in the tangibility dimension, which may reflect the material and living conditions of respondents. This is the area, which if better, could improve the perceived quality of health care and the level of patient satisfaction. Supporting respondents with better living facilities, which are helpful when it comes to caring for themselves or optimizing local conditions, could decrease the gap in the tangibility dimension.

Special attention should be paid to the fact that the smallest gap is in the empathy dimension. The perceived respect and understanding of a patient’s needs are close to the expectations. It reflects the high competences of personnel in this dimension and the high satisfaction of a patient with this dimension of care.

In the confidence dimension, the gap was relatively small, as well. The patients feel safe in this system of care. The competence of personnel, availability of the nurse, and courtesy of the personnel are very close to the patient’s expectations.

### Study Limitations

The study has several limitations. 

Firstly: The number of patients recruited is relatively low. The process of recruiting patients to the study was not randomized and the respondents live in one region of the country. This could be a limitation to the generalization of the results.

Secondly: The group of patients with pulmonological disorders was significantly bigger than the patients with neurological disorders. The analysis in the subgroups could be more valuable and may be more precise, which could be investigated in future research.

Thirdly: It must be noted that some surveys were completed by the respondents in the presence of another person (not a member of the therapeutic team). This fact was related to the physical possibilities of the examined patients. Twenty-one surveys were incomplete and deleted from the analysis. This is a relatively high proportion, which reflects the difficulties in communication with the ventilated patient.

Additionally, it is worth mentioning that only one method was used to measure the gaps between perceived and expected quality of services and patient’s satisfaction, which is the modified SERVQUAL questionnaire. A modification of the questionnaire was necessary. Nevertheless, in the tangibility dimension, more detailed investigations would be desirable. A deeper investigation of the tangibility dimension may increase the validity of the scale.

The patient’s perspective of the quality of services and patient’s satisfaction in HMV services need further investigations with an alternative method.

## 5. Conclusions

In conclusion, the level of satisfaction with health care among patients treated with HMV in the majority of investigated components is high. Moreover, the difference between perceived and expected quality of health care in the HMV system was relatively small in the opinion of the patients themselves. In this study, the biggest gap between the expectations and perceived quality is in the tangibility dimension, which may reflect the material and living conditions of respondents. Therefore, a deeper investigation of the tangibility dimension is needed. 

## Figures and Tables

**Table 1:** Group characteristics.

Variables		(N = 100)	
		N	%
Type of ventilation	NIV	71	71
	IV	29	29
Gender	male	65	65
	female	35	35
Marital status	married	64	64
	unmarried	36	36
Place of living	urban	51	51
	rural	49	49
Education	no/elementary	36	36
	vocational	28	28
	secondary/higher	36	36
Living condition	very good	24	24
	good	66	66
	difficult	10	10

NIV: Non-invasive ventilation; IV: Invasive ventilation.

**Table 2:** Comparison of perceived and expected service quality.

Assessed Components	*N*	Mean		Difference	Wilcoxon’s Test
		Perceived *P*	Expected *E*	*P-E*	*p*-value
Material status	100	3.55	4.52	−0.97	<0.001 ***
Accuracy of medical services	100	4.43	4.77	−0.34	<0.001 ***
Will to help	100	4.45	4.82	−0.37	<0.001 ***
Keeping calm and quiet	100	4.33	4.71	−0.38	<0.001 ***
Level of care	100	4.56	4.85	−0.29	<0.001 ***
Rapidity of help	100	4.54	4.85	−0.31	<0.001 ***
Response for patient’s requests	100	4.53	4.87	−0.34	<0.001 ***
Safety of the patient	100	4.40	4.84	−0.44	<0.001 ***
Courtesy of personnel	100	4.72	4.88	−0.16	0.003 **
Competence of personnel	100	4.74	4.87	−0.13	0.006 **
Availability of a physician	100	4.47	4.90	−0.43	<0.001 ***
Availability of a nurse	100	4.75	4.89	−0.14	0.009 **
Availability of a physiotherapist	83	4.51	4.88	−0.37	<0.001 ***
Respect for the patient	100	4.69	4.87	−0.18	<0.001 ***
Understanding for the patient’s needs	100	4.62	4.81	−0.19	0.001 **
Intimacy preservation	100	4.67	4.88	−0.21	<0.001 ***
Way of passing on information and ability to listen to the patient	100	4.64	4.87	−0.23	<0.001 ***

** *p* < 0.05; *** *p* < 0.001.

**Table 3:** Comparison of perceived and expected service quality for assessed dimensions.

Assessed Dimensions	*N*	Mean		Difference	Wilcoxon’s Test
		Perceived *P*	Expected *E*	*P-E*	*p*-value
Tangibility	100	3.55	4.52	−0.97	<0.001
Reliability	100	4.44	4.79	−0.35	<0.001
Responsiveness	100	4.54	4.86	−0.32	<0.001
Confidence	100	4.60	4.87	−0.27	<0.001
Empathy	100	4.66	4.86	−0.20	<0.001

**Table 4:** The distribution of values of tangibility dimension divided by groups created by demographical variables.

Demographical Variable		Tangibility Dimension				Test
	N	M	SD	Me	25–75%	*p*
Gender						0.662 ^(1)^
Male	65	3.55	0.92	4.0	3.0-4.0	
Female	35	3.54	0.82	4.0	3.0-4.0	
Marital status						0.546 ^(1)^
Married	64	3.56	0.92	4.0	3.0-4.0	
Unmarried	36	3.53	0.81	3.5	3.0-4.0	
Education						0.706 ^(2)^
No/elementary	36	3.42	1.00	4.0	3.0-4.0	
Vocational	28	3.61	0.83	4.0	3.0-4.0	
Secondary/higher	36	3.64	0.80	4.0	3.0-4.0	
Place of living						0.495 ^(1)^
Rural	49	3.47	0.96	4.0	3.0-4.0	
Urban	51	3.63	0.80	4.0	3.0-4.0	
Living conditions						0.0001 ^(2)^***
Very good	24	3.83 ^b^	0.76	4.0	4.0-4.0	
Good	66	3.62 ^b^	0.82	4.0	3.0-4.0	
Difficult	10	2.40 ^a^	0.70	2.5	2.0-3.0	

a,b: Post-hoc test designation; groups without a common designation differ significantly; *** *p* < 0.001; M: Mean; SD: Standard deviation; Me: Median; 25–75%: Quartile, ^(1)^ U Mann–Whitney, ^(2)^ ANOVA Kruskal–Wallis.

## Data Availability

The data that support the findings of this study are available from the corresponding author, (MKM), upon reasonable request.

## Supplementary Materials

The following are available online at https://www.mdpi.com/article/10.3390/ijerph18084048/s1, Table S1. Modified SERVQUAL questionnaire
