# Supplementary material for: Home Mechanical Ventilation: A Patient’s Perspective Survey Study
Source: Int J Environ Res Public Health. 2021 Apr 12;18(8):4048. doi: 10.3390/ijerph18084048 (PMC8069923; doi:10.3390/ijerph18084048)
Supplement: Supplementary file 1 [file ijerph-18-04048-s001.pdf]

Table S1. Modified SERVQUAL questionnaire.

Assessed Components	Expected	Perceived
**Tangibility**		
Assess the material situation in the family		
**Reliability**		
Assess the accuracy of medical services		
Assess the will to help the patient and the family in all medical problems		
Assess the personnel's level of keeping calm and quiet		
Assess the level of care		
**Responsiveness**		
Assess the rapidity of help		
Assess the response for the patient's requests		
**Confidence**		
Assess the safety of the patient		
Assess the courtesy of personnel		
Assess the competence of personnel		
Assess the availability of a physician		
Assess the availability of a nurse		
Assess the availability of a physiotherapist		
**Empathy**		
Assess the respect for the patient		
Assess the understanding of the patient's needs		
Assess intimacy preservation		
Assess the way of passing on information and the ability to listen to the patient		
